# Serum MMP-3/TIMP-1 ratio is a potential diagnostic marker for HIV-associated neurocognitive disorders

**DOI:** 10.21203/rs.3.rs-10474891/v1

**Published:** 2026-08-12

**Authors:** Sérgio Monteiro De Almeida, Indianara Rotta, Antony Liao, Bin Tang, Florin Vaida, Ana Paula de Pereira, Scott Letendre, Ronald J Ellis

**Affiliations:** 1 Universidade Federal do Paraná, Curitiba, Paraná, Brazil; 2 Department of Psychiatry, University of California, San Diego, CA, USA; 3 Division of Biostatistics and Bioinformatics, Department of Family Medicine and Public Health, University of California, San Diego, CA, USA; 4 HIV Neurobehavioral Research Center, University of California, San Diego, CA, USA; 5 Division of Infectious Diseases, Department of Medicine, University of California, San Diego, CA, USA; 6 Department of Neurosciences, University of California, San Diego, CA, USA

**Keywords:** HIV-1 subtypes, HIV-1C, cerebrospinal fluid (CSF), Metalloproteinases (MMPs), tissue inhibitors of MMPs (TIMPs)

## Abstract

Persistent central nervous system (CNS) inflammation and chronic immune activation contribute to HIV-1-associated neurocognitive disorders (HAND). Imbalances in the matrix metalloproteinases (MMPs) and tissue inhibitors of metalloproteinases (TIMPs) have been implicated in HAND pathogenesis. We hypothesized that MMP levels would be higher in the HAND group than in the other groups. This study assessed MMPs and TIMPs in the cerebrospinal fluid (CSF) and serum of people with HIV (PWH, n=68) and people without HIV (PWoH, n=19) categorized by neurocognitive performance using the Global Deficit Score (GDS) and HAND classification. Biomarker levels were compared using linear regression adjusted for plasma HIV RNA suppression and nadir CD4. The Benjamini–Hochberg method was used for multiple testing adjustment, and Spearman method (ρ) for correlation. Serum MMP-3/TIMP-1 ratio levels were elevated in the group with GDS >0.5 versus GDS <0.5 [0.048 (0.029; 0.068) versus 0.028 (0.018; 0.049), Cohen’s d 0.811 (0.274, 1.348), p BH-adjusted 0.036]. Working memory deficit was weakly correlated with MMP-2, MMP-9, TIMP-1, MMP-9/TIMP-1, and MMP-2/TIMP-2 in CSF (ρ 0.277–0.383), there was no correlation of these biomarkers in serum. The area under the receiver operating characteristic curve of MMP-3/TIMP-1 ratio for HAND diagnosis was 0.67, sensitivity was 64% (46%–79%), and specificity was 67% (45%–84%). Conclusion: Serum MMP-3/TIMP-1 ratio is a promising diagnostic biomarker of HAND, potentially eliminating the need for lumbar puncture and CSF collection.

## Introduction

Persistent central nervous system (CNS) inflammation and chronic immune activation play important roles in neuronal damage in HIV-associated neurocognitive disorders (HAND) (McArthur and Johnson, 2020; Ruhanya et al. 2021). Metalloproteinases (MMPs) are enzymes that significantly influence CNS inflammation by degrading the extracellular matrix (ECM), disrupting the blood-brain barrier (BBB), allowing immune cell entry, and activating inflammatory mediators, demyelination, and neuronal death. In addition to these detrimental activities, MMPs play a role in development and repair (Brkic et al., 2015).

MMP-9 is a hallmark in inflammatory diseases because of its tissue-damaging effects (Agrawal et al., 2006), related to inflammation-promoting processing of soluble proteins, including protease inhibitors (Liu et al., 2000), chemokines (Van den Steen et al., 2000), and cytokines (Heissig et al., 2002; Nelissen et al., 2003; Yu and Stamenkovic, 2000). MMP-2 has anti-inflammatory and homeostatic functions; it presumably inactivates inflammatory chemokines (McQuibban et al., 2000).

The balance between MMPs and their respective tissue inhibitors (TIMPs) is critical for maintaining brain homeostasis, and is often disrupted in diseases (Mannello and Gazzanelli, 2001). In addition to inhibiting the activity of several MMPs, the TIMP family displays several pleiotropic functions (growth factor-like activity, suppression of cell death, mitogenic activity, and modulation of cell morphology), which are required for the activation of some MMPs. Assuming conflicting paradoxical roles (Yong et al., 2001; Mannello and Gazzanelli, 2001).

MMPs and TIMPs dysregulation in peripheral blood (PB) and cerebrospinal fluid (CSF) is associated with HAND pathogenesis. This imbalance is related to altered profiles of inflammatory cytokines, chemokines, and monocyte activation. In addition, plasma from people living with HIV (PWH) directly induced integrity disruption in an in vitro BBB model, leading to increased BBB permeability and transmigration of monocyte macrophages (Xing et al., 2017).

To investigate the relationship between MMPs and TIMPs in the CSF and serum, and neurocognitive performance in PWH, we compared MMP and TIMP levels and the associated ratios between groups categorized according to neurocognitive status. We hypothesized that MMP and TIMP levels in the CSF and serum would be higher in individuals with HAND than in those without HAND. Furthermore, we described the diagnostic clinical performance characteristics (DCPC) of MMP and TIMP in the CSF and serum for the diagnosis of HAND.

## Methods

### Standard protocol approvals, registrations, and patient consents

This study was approved by the UCSD Institutional Review Board (IRB), Hospital de Clínicas–Universidade Federal do Paraná IRB, and CONEP. All participants signed informed consent forms approved by institutional review boards in the United States and Brazil. CSF samples were collected as part of a NIMH-funded study (R21 MH076651-01).

### Study design and participants

This study was a cross-sectional survey analyzing stored blood and CSF samples. Paired CSF and serum samples were collected from PWH (n=68) and HIV-negative controls (PWoH, n=19). PWH were recruited from the Hospital de Clínicas da Universidade Federal do Paraná (HC-UFPR), Brazil. The exclusion criteria were opportunistic CNS infections, loss of consciousness for >30 min, non-HIV-related neurological injury, or disorders (e.g., epilepsy, stroke, and developmental delay). All volunteers underwent serological testing to confirm their HIV status before enrollment (de Almeida et al., 2013).

### CSF and blood samples

CSF samples were collected exclusively for research purposes through lumbar puncture (LP) performed using atraumatic spinal needles and aseptic techniques. CSF and serum samples were collected in polypropylene tubes to avoid adherence of the proteins to the tube walls. Serum was separated from cells immediately after collection. Just after processing, CSF and serum aliquots were frozen and appropriately stored at −80°C, at HC-UFPR, until the day of the assays, and were not subjected to freeze-thaw cycles. The average storage duration was four years (Sulik et al., 2008; Jonsson et al., 2018). To prevent possible interference with the determinations, no preservative solution was added to the samples.

For participants with clinically resistant infections, the HIV subtype was genotyped using polymerase sequences, whereas envelope glycoprotein sequences were used for all other participants. Genotyping revealed that 27 individuals were infected with HIV-1B, and 40 were infected with non-B HIV-1 subtypes (C, n=26; BF, n=10; BC, n=1; CF, n=1; F, n=2). The subtype of one participant could not be determined (de Almeida et al., 2013). For a subset of 40 participants, transactivator of transcription (Tat) sequencing was performed for amino acid variants Tat C30C31 (n= 22) and Tat C30S31 (n= 18). The Tat C30S31 amino acid variant is predominantly present in HIV-1C Tat (de Almeida et al., 2021).

Nineteen subjects, with sex- and age-comparable distribution, were recruited at the HIV Neurobehavioral Research Center, University of California, San Diego (HNRC-UCSD) for the PWoH control group. According to the National Commission of Ethics in Research (CONEP, Brazil), lumbar puncture (LP) cannot be performed on PWoH in Brazil. These subjects had no neurological comorbidities or cognitive complaints and had negative serological tests for HIV, hepatitis C, and syphilis. The CSF inclusion criteria for the PWoH group were white blood cell (WBC) count ≤ 5 cells/mm^3^, total protein ≤ 45 mg/dL, and glucose ≥ 55 mg/dL.

### Laboratory methods

#### Quantification of CSF and serum biomarkers

CSF samples were collected for research purposes via LP using atraumatic spinal needles under aseptic conditions. MMP-2, −3, and −9 levels were quantified using multiplex bead suspension array immunoassays (EMD Millipore, Billerica, MA, USA); the lower limit of detection (LLOD) is 200, 14, and 2 p/mL, respectively. TIMP-1 and −2 were measured using an electrochemiluminescence assay (MesoScale Discovery, Rockville, MD, USA); the LLOD is 20 pg/mL for each. All samples were assayed concurrently in duplicate, according to the manufacturer’s instructions. The acceptable coefficient of variation between duplicates was <20%. When the results were below the MDC set by the kit manufacturer, the MDC was used in the statistical analysis.

To enhance the specificity of the biomarkers, MMPs and TIMPs CSF/serum quotients (Q) and MMP-2, MMP-3, and MMP-9 index (Idx) were calculated. Idx was derived from the molecular weights (MW) and hydrodynamic radii (Stokes radii). The MW of MMP-2, −3, and −9 are 72, 54, and 92 kDa, respectively, and their hydrodynamic radii are 45.00, 29.30, and 44.10 Å, respectively. MMP Idx was calculated using the following formula: MMP Idx = (CSF MMP × serum albumin) / (serum MMP × CSF albumin). The index was not calculated for TIMP-1 and TIMP-2 as they are small molecules (MW of 28 and 22 kDa, with hydrodynamic radius of 34 and 28.06 Å, respectively). Small molecules cross the blood–CSF barrier independent of its integrity because diffusion through an intact barrier may occur along a concentration gradient (Reiber et al., 2012). The reference values for MMP-2, −3, and −9; TIMP-1 and −2; and MMP Idx were calculated as means ± 2 standard deviations in the PWoH group, or the receiver operating characteristic (ROC) curve.

Albumin levels in the CSF and serum were quantified using nephelometry (Dade Behring BNII; Deerfield, IL, USA) with antiserum N human albumin (Dade Behring BNII).

### Neurobehavioral assessments

All PWH underwent neuropsychological (NP) assessments, as previously described (Heaton et al, 2004; de Almeida et al., 2013). The NP test battery assesses seven domains (executive function, motor performance, verbal fluency, recall, working memory, learning, and speed of information processing). It is composed of 18 individual NP measures widely used to study HIV infection in previous studies in English- and non-English-speaking countries. Instruments not already validated for use in Brazil were translated into Brazilian Portuguese, back translated into English, and reviewed by several native Brazilian Portuguese speakers to ensure cultural and linguistic appropriateness (de Almeida et al., 2013).

Demographic corrections for each NP test were performed using data from 48 HIV−controls in Brazil. The norms were statistically derived by methods previously described (de Almeida et al., 2013). Briefly, raw scores for the individual tests were converted to normally distributed scaled scores, with a mean of 10 and standard deviation of 3. The scaled scores were then converted to T-scores (mean=50, SD=10) with regression-based adjustments for age, years of education, and sex. Subjective neurocognitive difficulties were assessed using the Patient Assessment of Own Functioning Inventory (Chelune et al., 1986). HAND was categorized based on cognitive and functional impairments on: neuropsychological normal (NP-NML, n= 24), asymptomatic neurocognitive impairment (ANI, n= 29), mild neurocognitive disorder (MND, n= 2), and HIV-associated dementia (HAD, n= 5) (Antinory et al., 2007).

Additionally, the global deficit score (GDS) method was used to classify the overall NP impairment status, as previously described. The GDS summarizes the number and severity of the neurobehavioral deficits across the entire test battery. A GDS cutoff of <0.50 was used to classify overall NP impairment (Carey et al., 2004; de Almeida et al., 2013). To assess domain-specific neurocognitive performances, domain deficit scores were calculated by averaging individual test deficit scores within each domain.

### Data analyses

The results were presented as medians and interquartile ranges (IQRs) or numbers and percentages (%), as appropriate. Comparisons of the demographic, clinical, and laboratory characteristics of HIV infection between the PWH and PWoH groups were performed using the independent samples t-test for continuous variables and Fisher’s exact test for binary and categorical variables.

CSF and serum biomarker values were log_10_-transformed to improve the normality of data distribution. Comparisons were performed to assess the differences of MMPs, TIMPs, MMP/TIMP ratio, and index in CSF, serum, and CSF/serum (Q) between the GDS<0.50 and GDS≥0.50 groups using linear regression, controlling for age. Comparisons across multiple groups (PWoH and the HAND subgroups in PWH) were performed using analysis of covariance (ANCOVA) and pairwise comparisons with Tukey’s Honestly Significant Difference (HSD) test, adjusting for HIV RNA and nadir CD4. Multiple testing correction was performed using the Benjamini-Hochberg (BH) procedure. The mild neurocognitive disorder (MND) group was not included in the analyses due to the small number of cases (n = 2).

Differences were considered statistically significant at 5% alpha level. Standardized regression coefficients (analogous to Cohen’s d) and their 95% confidence intervals (CIs) were reported.

Spearman’s rank-order correlation coefficients (ρ) were used to evaluate associations between biomarkers and global and domain-specific deficit scores in the PWH group.

### Diagnostic Clinical Performance Characteristics

We evaluated the DCPC of CSF, serum MMP and TIMP, and their associated ratios in predicting HAND. The DCPC for the diagnosis of HAND was calculated using as clinical measurements, i.e. GDS or the HAND classification.

The following DCPC were calculated: sensitivity, specificity, accuracy (efficiency), combined error, positive and negative predictive values (PPV and NPV), Youden index, positive and negative clinical utility index (CUI+ and CUI−), positive and negative likelihood ratios (LR+ and LR−), diagnostic odds ratio (DOR), and Matthews correlation coefficient (MCC) (Galen and Gambino, 1975; McGee 2002; Glas et al. 2003; Akobeng 2007a; Matthews 1975). The CUI values were classified as follows: excellent, ≥ 0.81; good, ≥ 0.64; fair, ≥ 0.49; poor, ≤ 0.49; and very poor, ≤ 0.36 [Mitchell, 2008; Mitchell, 2011]. An LR+ value ≥10.0 indicates that a positive test almost confirms the disease, a value of ~6.0 indicates that the disease was present, and a value of ~1.0 indicates that the test was not able to show whether the disease was present or not. An LR+ value ≤0.1 indicates that the disease was practically absent [McGee, 2002; Akobeng, 2007a]. The DOR indicates the ability of the test to discriminate between the presence and absence of a condition. A higher DOR value signified better test performance, while a DOR of 1 suggests that the test offers no useful information. The DOR itself is independent of disease prevalence in the population, unlike the PPV and NPV. The MCC is a value between −1 and +1 (Matthews 1975). Post-test probabilities were calculated using Bayes’ theorem with pre-test probabilities and likelihood ratios plotted on Fagan’s nomogram (Fagan 1975; Sackett and Haynes 2002). A ROC curve was used to determine the cutoff value associated with the highest distinction between the HAND groups and normal neuropsychological function. The Youden index (J) was calculated for all points on the ROC curve. The maximum value of the index was used as a criterion to select the optimal cut-off point for a diagnostic test, the area under the ROC curve (AUC) was analyzed, and the difference between the curve and the baseline (random line) was calculated (Akobeng 2007a; 2007b).

## Results

The 87 participants had a mean age of early 40 years, with higher education in PWoH than PWH (mean 12 *vs* 8 years, *p* = 0.001). Most of PWH were virally suppressed (55.9% plasma viral load ≤50 copies/mL); 80.9% had an AIDS diagnosis and 94.4% adherence. The median nadir and current CD4+ T cell counts were 90 and 369 cells/mm^3^. Demographic, clinical, and laboratory characteristics of PWH and PWoH were summarized and compared. (Suppl. Table 1).

### Comparison between groups categorized by the GDS and HAND classification

Serum MMP-3/TIMP-1 ratio was higher in the group with GDS ≥0.50 than that in the group with GDS <0.50 (median (interquartile), 0.048 (0.029; 0.068) *vs* 0.028 (0.018; 0.049), the BH-adjusted *p* =0.036), with a large effect size (Cohen’s d=0.811, 95% CI = 0.274 – 1.348). The group with GDS ≥0.50 had marginally higher serum MMP-3 and MMP-9/TIMP-1 than the group with GDS <0.50 (*p*=0.031 and 0.037, BH-adjusted *p*= 0.098 for both). A higher ratio of MMP to TIMP indicates lower inhibition and active inflammation. Q_MMP-3_ was marginally different between the two groups (*p*=0.018, BH adjusted *p*=0.120), supporting the finding that the inflammatory reaction is higher in the PB than in the CNS and is associated with impaired global cognition. In CSF, the groups were comparable (Table 1).

CSF MMP-2, MMP-9, MMP-9/TIMP-1, and MMP-2/TIMP-2 levels were higher in the HAD group than in the PWoH group (*p*=0.0029–0.0098) (Table 2). CSF MMP-2 had the largest effect (d= 1.754, 95% CI (0.63, 2.878). CSF MMP-2, MMP-9, MMP-2/TIMP-2, and MMP-3/TIMP-1 ratio were higher in the PWH NP-NML group than in the PWoH group (*p* <0.0001 to 0.0067) (Table 2). Cohen’s d (95% CI) for the significant difference between the groups is shown in the Suppl. Table 2; MMP-2 had the highest effect size (d=1.086, 95% CI (0.422, 1.75). This difference may be due to HIV infection rather than the HAND. Serum TIMP-1 levels were higher in the PWH NP-NML and asymptomatic groups than in the PWoH group (*p*=0.0086 and 0.0551; d=0.910 (0.278; 1.542) and 0.617 (0.025; 1.208), respectively (Table 3).

In both CSF and serum, the biomarkers didn’t differ across the three HAND subgroups (Tables 2 and 3).

Among PWH, MMP-2 and −3 indices were significantly different in the asymptomatic versus the PWH cognitively normal group (*p*=0.0089 and *p*=0.0061, respectively), with a high effect size (Cohen’s d=−0.747, 95% CI (−1.307; −0.188) and −0.805 (−1.367; −0.243), respectively) (Suppl Table 3).

More disparities in MMP and TIMP levels in the CSF, serum, and associated formulas were identified between the HAND subgroups and PWoH (Tables 2–4). Cohen’s d (95% CI) was shown in Suppl Tables 2 and 3 if the difference between groups was significant.

### Diagnostic Clinical Performance Characteristics

DCPC were calculated for serum MMP-3/TIMP-1 ratio, MMP-2 Idx and MMP-3 Idx to diagnose HAND (Suppl Table 4). These predictors were selected because they differed significantly between the groups (Tables 1 and 4). Using serum MMP-3/TIMP-1 ratio, DCPC were calculated for distinguishing the PWH group with GDS≥0.5 (n= 36) from the group with GDS<0.5 (n=24); using MMP-2 and MMP-3 Idx, the asymptomatic group (n= 29) was distinguished from the group with normal neurocognitive performance (n=24). The AUC of the three HAND predictors is shown in Figure 3.

### Spearman’s correlations of MMP and TIMP with GDS and domain-specific cognitive deficits

Working memory deficit was weakly correlated with CSF MMP-2 (*ρ*= 0.349 (0.094–0.561), p=0.007), MMP-9 (*ρ*=0.383 (0.133–0.587), p=0.003), TIMP-1 (*ρ*=0.277 (0.015–0.504), p=0.034), MMP-9/TIMP-1 (*ρ*= 0.359 (0.106–0.569), p=0.005), and MMP-2/TIMP-2 (*ρ*=0.284 (0.022–0.509, p=0.030) (Figure 2). There was no correlation between CSF MMP, TIMP, or the MMP/TIMP ratio and other domain-specific or global deficits. No correlation was identified between working memory deficit and serum MMP, TIMP, or the MMP/TIMP ratio.

There was a weak positive correlation of GDS with serum MMP-9/TIMP-1 (*ρ*= 0.268, p=0.039) but no correlation with CSF MMP, TIMP, and the MMP/TIMP ratio (Suppl. Table 4). Significant correlations of specific cognitive domains with MMP, TIMP, or MMP/TIMP in serum, CSF/serum, and Index are shown in Suppl. Tables 4 and 5.

## Discussion

In this study, we quantified MMP-2 and MMP-9 (gelatinase A and B, respectively), MMP-3 (stromelysin-1), and their respective tissue inhibitors, TIMP-1 and TIMP-2, across groups categorized according to neurocognitive status. Serum MMP-3/TIMP-1 levels, but not CSF levels, were significantly higher in the group with cognitive impairment than in the group without cognitive impairment, as indicated by GDS. This may indicate a peripheral-driven process influencing BBB permeability, potentially mediated by MMP-3 proteolytic activity. Elevated serum MMP-3/TIMP-1 ratio levels are consistent with HIV being a systemic disease, disrupting tight junction proteins (occludin and claudins) and ECM, compromising BBB integrity, and facilitating the influx of peripheral factors into the CNS, contributing to neuroinflammation and neurodegeneration. Conversely, the absence of this imbalance in CSF implies that the CNS compartment may have compensatory mechanisms. This peripheral biomarker signature reflects systemic contributions to cognitive decline. As such, further investigation into the MMP and TIMP axis as a diagnostic or therapeutic target for preserving BBB function and mitigating neurodegenerative processes is warranted.

In turn, when comparing PWH with PWoH, our group previously described that CSF MMP-2 and MMP-9 levels and their ratios with respective TIMPs were higher in PWH than in PWoH. In contrast, serum MMP levels were comparable between the groups, suggesting a compartmentalized inflammatory response in the CNS in PWH (de Almeida et al., 2026). The increased influx of MMPs and TIMPs from PB associated with the disruption of the ECM overwhelms the intrathecal inflammatory response. Additionally, several inflammatory proteins, as well as HIV-1 Tat present in PB and CSF, contribute to enhancing BBB dysfunction and the CNS inflammatory response. This, combined with intrathecal MMP production, may explain the elevated CSF levels of MMPs and their ratios with respective TIMPs in PWH compared to PWoH (Yong et al., 2001; de Almeida et al., 2026).

Serum MMP-3/TIMP-1 ratio is a promising diagnostic tool for HAND, particularly in areas with limited access to or restrictions on LP. A higher ratio of MMP to TIMP indicates lower inhibition of MMP activity and active inflammation. Based on the DCPC metrics, the serum MMP-3/TIMP-1 ratio acts as a weak-to-moderate supplementary diagnostic biomarker for HAND. It is not suitable as a standalone diagnostic tool because of its low accuracy and poor ability to discriminate between HAND and non-HAND patients. A positive result increases the likelihood of a disorder; however, a negative result does not rule this out. The clinical utility of the test was moderate and should be interpreted with caution. It should not be used to rule out HAND, as it showed low NPV and high negative LR. Therefore, more studies are necessary.

MMP-2 Idx and MMP-3 Idx were significantly different in the PWH ANI versus the NP-NML group. The MMP Idx is an association of biomarkers in CSF and serum, with the aim of enhancing the diagnostic power of isolated biomarkers. For MMP-2 Idx and MMP-3 Idx, the AUC were 0.71 and 0.72, respectively, indicating a diagnostic test that is acceptable with good discrimination (Çorbacıoğlu and Aksel, 2023). These indices are potentially useful tools for the diagnosis of HAND, particularly for discriminating ANI from NP-NML, which is challenging. This differential diagnosis is crucial for early detection and prediction of HAND. Longitudinal studies have demonstrated that PWH with ANI have a significantly higher risk of progressing to symptomatic neurocognitive impairment (i.e., MND and HAD), even in patients who are virally suppressed by combination antiretroviral therapy (Grant et al., 2014). MMP indices are a promising tool, particularly when MMP-2 is used for increased accuracy. The MMP-2 Idx is a moderate tool for identifying CNS immune activation related to HAND. With an accuracy of 66% and moderate LR+, it is best used as a supplementary tool in combination with NP tests and other markers, rather than as a standalone diagnostic test. A positive result warrants further neurological evaluation. However, a negative result cannot exclude the presence of mild neurocognitive dysfunction.

MMP-3 index acts as a modest to weak biomarker for HAND. Because of its low sensitivity and specificity, this may be useful when combined with other strong markers to increase the overall AUC. The MMP-3 Idx has a limited diagnostic value for HAND. Although it has a good discriminatory power, its accuracy and clinical utility are moderate. A positive result increased the likelihood of a disorder, whereas a negative result was less informative. Overall, the MMP-3 index is a biomarker with limited diagnostic value for HAND, and its results should be interpreted with caution.

The three biomarkers, serum MMP-3/TIMP-1 ratio, MMP-2 Idx, and MMP-3 Idx, are not suitable as stand-alone diagnostic tools, but they may offer some value when combined with other tests or markers.

Within this research, serum TIMP-1 levels were significantly higher in the NP-NML group and marginally higher in the ANI group than in the PWoH group, with no differences in the other groups, or in the CSF and the associated ratios. TIMP-1 is a tissue inhibitor of MMP-3 and MMP-9. This increase is in response to the elevation in MMP, particularly MMP-9, which is corroborated by the significantly higher levels of Q_MMP-9_ in the NP-NML and ANI groups than in the PWoH group, as well as the dysregulation of MMP-3 Idx and serum MMP-3/TIMP-1 ratio. Other studies have shown that within the context of HAD, TIMP-1 expression by astrocytes is likely to be altered within the CNS, and MMP dysregulation in HAD has already been established (Conant et al., 1999; Ghorpade et al., 2001). MMP-9 is induced by cytokines, including TNF-α (Al-Roub et al., 2023). IL-1 expression is up-regulated in HAD (Vitkovic et al., 2000) and induces TIMP-1 in astrocytes, stimulating astrocyte proliferation (Wilczynska et al., 2006, Ogier et al., 2005). TIMP-1 is critical in the pathogenesis of CNS diseases, owing to its dynamic regulation of development. Mononuclear phagocytes that are infected with viruses and/or are immune-activated provide a logical source to drive inflammation and consequently upregulate TIMP-1 (Gardner and Ghorpade 2003).

In this study, working memory deficits were weakly correlated with MMP-2, MMP-9, TIMP-1, MMP-9/TIMP-1, and MMP-2/TIMP-2 in the CSF; however, there was no correlation between these biomarkers in the serum. The association of a biomarker with a specific but not global cognitive deficit could indicate localized BBB dysfunction or a low-grade chronic inflammatory process. While GDS serves as a global analysis tool, analyzing specific memory domains is crucial when total GDS scores lack statistical significance. A significant deficit in one domain may be masked by strong performance elsewhere, whereas domain-level analysis offers higher sensitivity to early-stage impairment. That is particularly relevant in our cohort, which has a predominance of asymptomatic over HAD cases (29/5, respectively). Therefore, even if the overall GDS score is not significant, PWH may still exhibit early memory loss.

This study has some potential limitations: (a) most of the participants were receiving ART, but some were not on antiretroviral treatment. We attempted to overcome this limitation by adjusting for the confounding effect of plasma HIV viral load in multivariable analysis. Nonetheless, HIV-1B and HIV-1C were found to be comparable in terms of the CSF-to-plasma HIV RNA ratio. (b) The cross-sectional design of the study represents a limitation. A longitudinal study may provide greater insight into the prediction of HAND development in individuals without apparent symptoms. (c) The small sample size is a limitation mainly for subgroup analyses. However, despite this, the medium to large effect size indicates meaningful results, suggesting the findings are considerable and have practical importance. The MND group was not included in the analyses due to the small number of cases (n = 2). (d) the small number of sample limits the interpretation of the DCPC.

(e) This study did not quantify other members of the MMP family, such as MMP-7, which has been associated with HAND (Conant et al., 1999; Singh et al., 2016). (f) The PWoH group was from North America (UCSD), whereas the PWH group was from Brazil, as the National Commission of Ethics in Research (CONEP, Brazil) does not permit LP in healthy participants. This mismatch can introduce confounding factors; we attempted to mitigate this by selecting comparable samples on age and sex.

Additionally, the genetic background in southern Brazil is mostly caucasian, similar to that in the USA.

This study adds to the literature by calculating the DCPC of MMP and TIMP for the diagnosis of HAND. Serum MMP-3/TIMP-1 ratio is a promising diagnostic biomarker for HAND, potentially eliminating the need for LP and CSF collection. CSF analysis is the most important method for evaluating the impact of HIV infection on the brain. Since CSF analyses require an LP, it would be helpful if this could be circumvented by the assessment of blood biomarkers. We introduced the concept of the MMP Idx, which analyzes four biomarkers in the CSF and serum. This index demonstrated promising applicability. However, further studies are required to test and validate their applicability.

Defining biomarkers for HAND is crucial for early and objective diagnosis, tracking disease progression, differentiating HAND from other conditions such as Alzheimer’s disease, and monitoring treatment effectiveness. Current HAND diagnosis relies heavily on cognitive testing; however, biomarkers offer objective biological signs of brain injury and inflammation, providing a clearer picture than just symptoms or viral load.

## Supplementary Files

This is a list of supplementary files associated with this preprint. Click to download.

- SupplTable1.docx
- Suppl.Table2.docx
- Suppl.Table3.docx
- SupplTable4.docx
- Suppltable5.docx
- Suppltable6.docx

**Suppl. Table 1. Demographics, clinical, and laboratory characteristics of HIV infection in PWH and PWoH** Values represent the median (IQR) or number of cases (%) as appropriate. Comparisons were performed using an independent samples t-test for continuous variables and Fisher’s exact test for binary and categorical variables.$$

PWH, people with HIV; PWoH, people without HIV; CART, combination antiretroviral therapy; CSF, cerebrospinal fluid; CPE, CNS Penetration-Effectiveness rank; GDS, global deficit score; RBC, red blood cells; WBC, white blood cells; TP, total proteins. CSF samples were collected purely for research purposes through lumbar puncture, performed using atraumatic spinal needles under aseptic techniques. Total CSF protein, glucose, and WBC counts were quantified using standard laboratory methods. HIV RNA levels in the serum and CSF were quantified using a branched DNA assay (Siemens) with a nominal limit of detection at 50 copies/mL. Peripheral blood current CD4 and CD8 counts were quantified by flow cytometry (FACSCalibur-Multitest), while nadir CD4 level information was extracted from the medical records. HIV RNA levels in plasma and CSF were measured using a branched DNA assay (VERSANT^®^ HIV-1 RNA 3.0 bDNA Kit, Siemens Healthcare Diagnostics, Tarrytown, NY, USA) with 1 mL of CSF or plasma. The assays were performed immediately after sample collection. Samples with HIV RNA levels <50 copies/mL were recorded as being below the detection limit. Data are presented as log10 values.

**Suppl. Table 2. Comparisons of CSF MMP and TIMP between the HAND groups and PWoH** Values represent the Cohen’s d (95%CI), displaying only significant pairwise comparisons. MMP, metalloproteinases; TIMP, tissue inhibitor of metalloproteinases; CSF, cerebrospinal fluid.

HAND was categorized as four groups: (A) neuropsychologically normal- NP-NML, (B) asymptomatic neurocognitive impairment- ANI, (C) HIV-associated dementia- HAD. The mild neurocognitive disorder (MND) group was not included in the analyses due to the small number of cases (n = 2).

PWoH (n=19); A. NP-NML (n=24); B. ANI (n= 29); C. HAD (n=5)

**Suppl. Table 3. Comparisons of MMP and TIMP in CSF/serum quotient (Q) and indexes between the HAND groups and PWoH** Values represent the Cohen’s d (95%CI), displaying only significant pairwise comparisons. MMP, metalloproteinases; TIMP, tissue inhibitor of metalloproteinases; CSF, cerebrospinal fluid. HAND was categorized on: (A) neuropsychologically normal- NP-NML, (B) asymptomatic neurocognitive impairment- ANI, (C) HIV-associated dementia- HAD. The mild neurocognitive disorder (MND) group was not included in the analyses due to the small number of cases (n = 2).

PWoH (n=19); A. NP-NML (n=24); B. ANI (n= 29); C. HAD (n=5)

MMP Index = (CSF MMP × albumin serum) / (serum MMP × albumin CSF).

**Suppl Table 4. Clinical performance characteristics of MMP-3/TIMP-1 ratio in serum, MMP-2 and −3 indexes to the diagnostic of HIV-Associated Neurocognitive Disorder** For serum MMP3/TIMP the clinical performance characteristics were calculated with the PWH group with GDS≥0.5 (n= 36) compared with PWH group with GDS<0.5 (n=24); for MMP2 and MMP-3 Idx, the groups of the HAND classification were compared, asymptomatic (n= 29) versus normal neurocognitive evaluation (n= 24).

[1] The cut-off point was defined with the ROC curve (figure).

TP, true positive; TN, true negative; FP, false positive (Type I error); FN, false negative (Type II error); PPV, positive predictive value; NPV, negative predictive value; LR, likelihood ratio; DOR, diagnostic odds ratio (DOR= LR+/LR-, the higher the DOR value, the better the test); CUI, clinical utility index; MCC, Matthews correlation coefficient; AUC, area under the ROC curve. Combined error = (FP+FN)/Total tested; posterior odds = [probability/(1-probability)]× LR $\text{MCC=}\frac{TP\times TN-FP\times FN}{\sqrt{\left(TP\times FP)(TP\times FN)(TN\times FP)(TN\times FN\right)}}$, the MCC returns a value between −1 and 1 where: +1 describes a perfect prediction; 0 unable to return any valid information (no better than random prediction).

Youden Index ranges between 0 and 1, with 0 values indicating that a diagnostic test gives the same proportion of positive results for groups with and without the disease. Values of 1 indicate that there are no false positives or false negatives.

**Suppl. Table 5. Correlation of serum MMPs and TIMPs with GDS, cognitive domains deficits, and Beck depression inventory score** Values represent correlation coefficients (*ρ*) estimated using Spearman’s rank-order method, and p values. Only significant correlations are observed. PWH, people with HIV; CSF, cerebrospinal fluid; MMPs, metalloproteinases; TIMPs, tissue inhibitor of metalloproteinases; GDS, global deficit score; SIP, speed of information processing; BDI, Beck depression inventory

**Suppl. Table 6. Correlations of MMP and TIMP CSF/ serum quotients and MMP indexes with GDS, cognitive domains deficits, and Beck depression inventory score** Values represent correlation coefficients (*ρ*) estimated using Spearman’s rank-order method, and p values. Only significant correlations are observed. PWH, people with HIV; CSF, cerebrospinal fluid; Q, CSF/ serum quotients; Idx, index; MMPs, metalloproteinases; TIMPs, tissue inhibitor of metalloproteinases; GDS, global deficit score; SIP, speed of information processing; BDI, Beck depression inventory

## Figures and Tables

**Figure 1.**
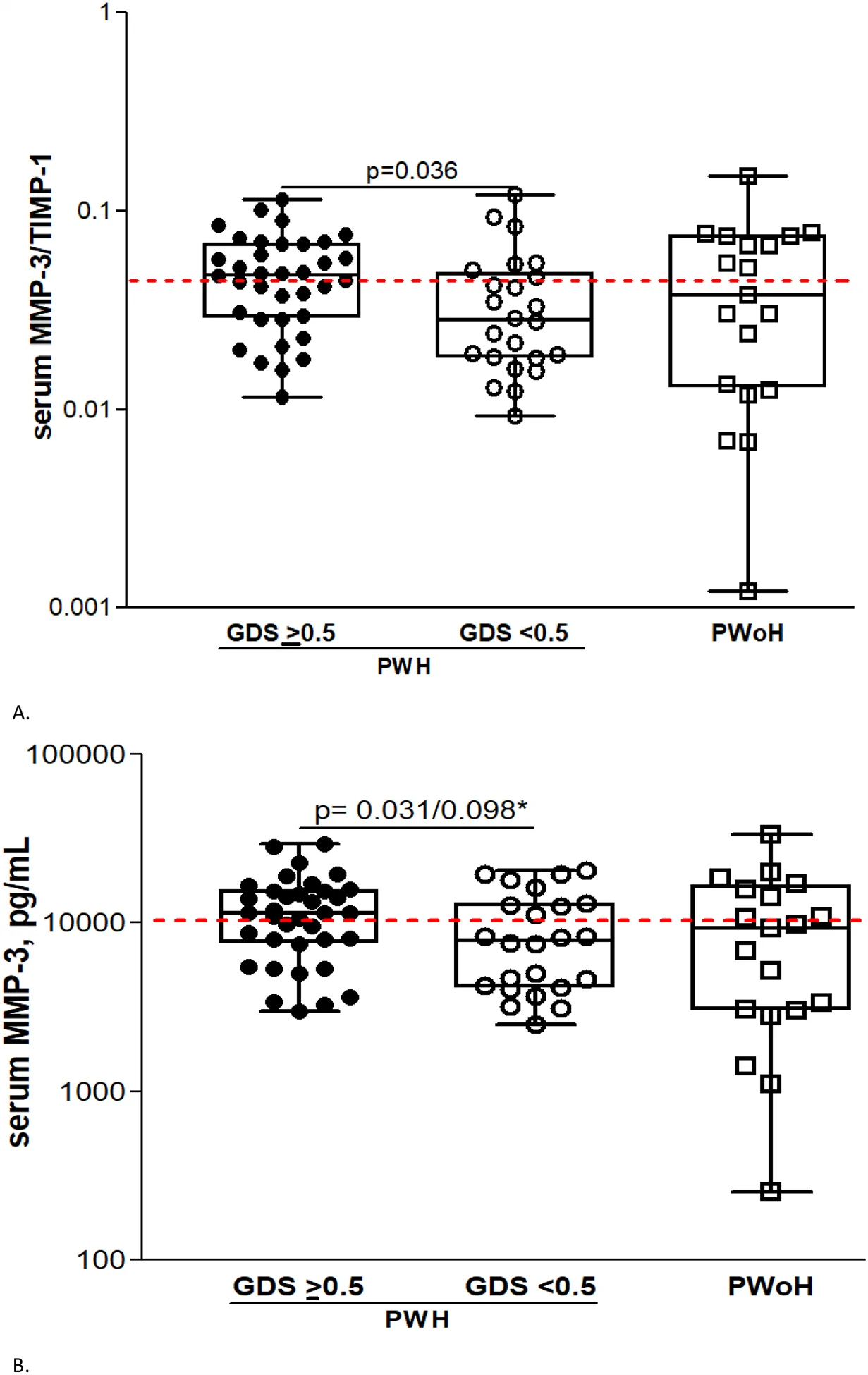

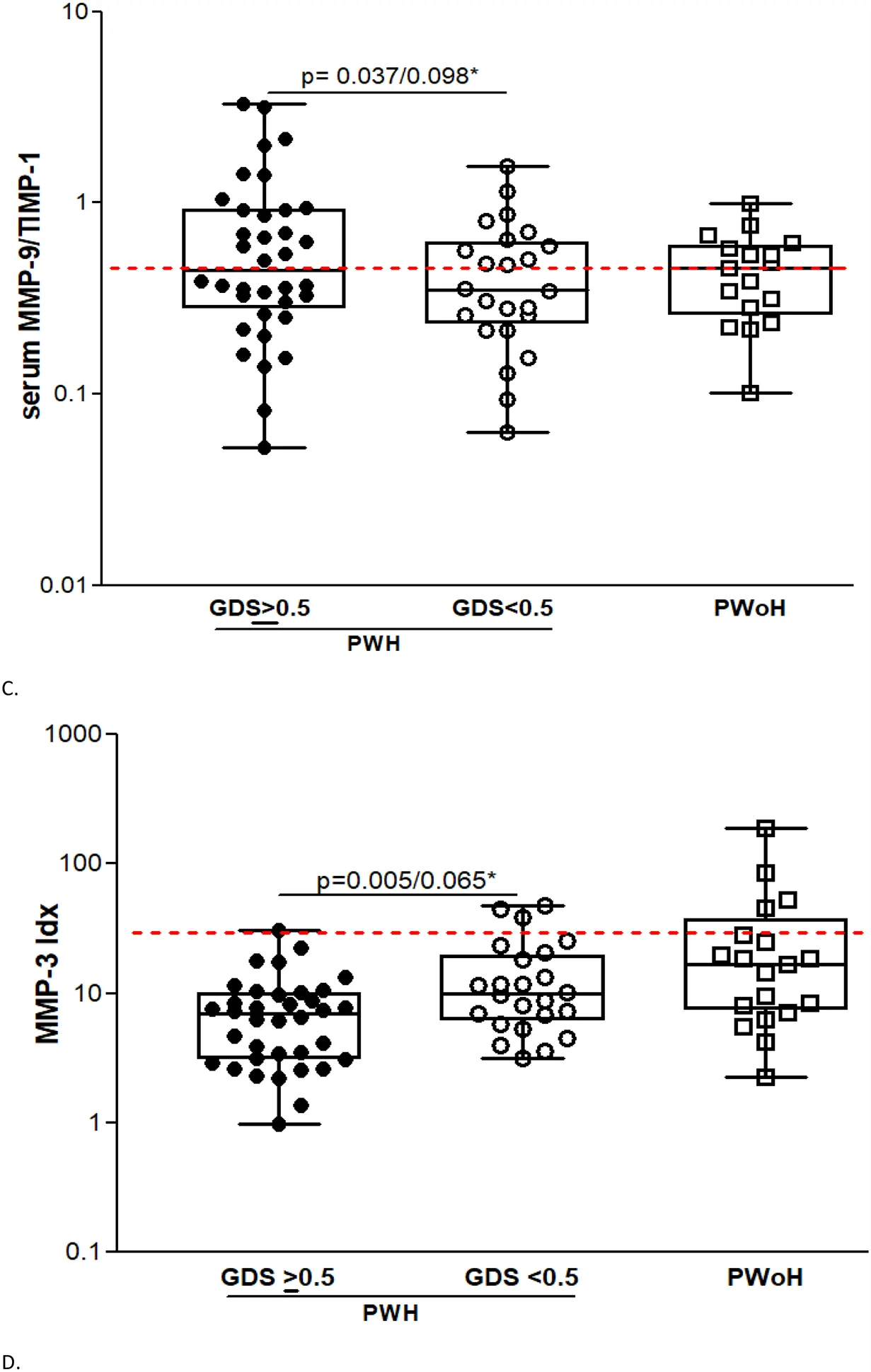
Levels of MMP and TIMP in CSF and serum, by groups of PWH categorized according to the GDS A. serum MMP-3/TIMP-1; B. serum MMP-3; C. serum MMP-9/TIMP-1; D. MMP3 Idx MMP, metalloproteinases; TIMP, tissue inhibitor of metalloproteinases; CSF, cerebrospinal fluid; GDS, global deficit score. Boxes show the median and interquartile range (IQR), and whiskers indicate the maximum and minimum values. Dots indicate the number of individuals in each group. The dashed red line indicates reference value (mean of PWoH group), serum MMP-3/TIMP-1 0.046 ± 0.075; serum MMP-3 [pg/mL] 9828.000 ± 16956; serum MMP-9/TIMP-1 0.456 ± 0.454; MMP3 Idx. 29.53 ± 86.10 * p-value significant before multiple comparison adjustment with the Benjamini–*Hochberg* procedure (BH), not significant after BH. MMP Index = (CSF MMP × albumin serum) / (serum MMP × albumin CSF).

**Figure 2.**
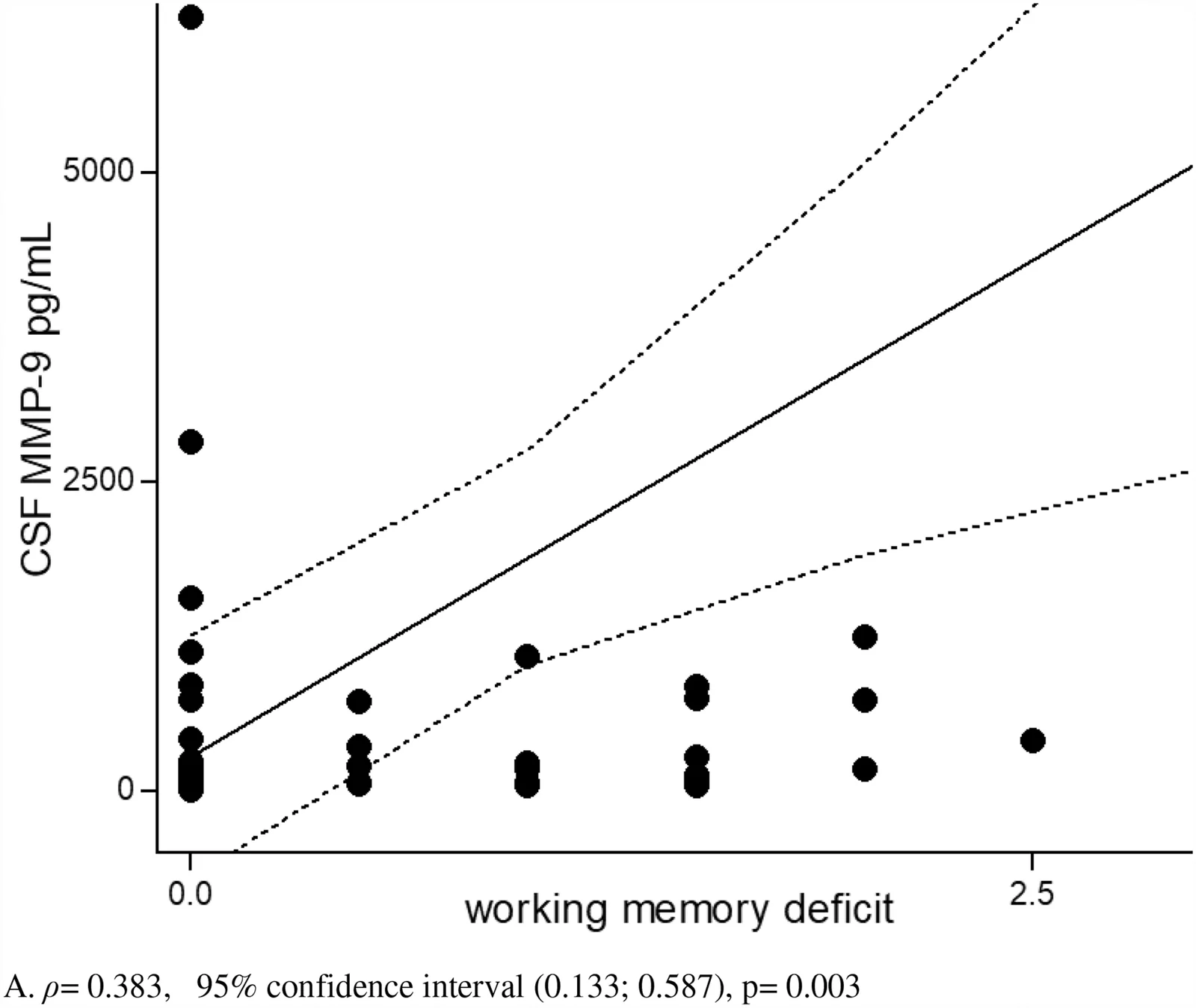

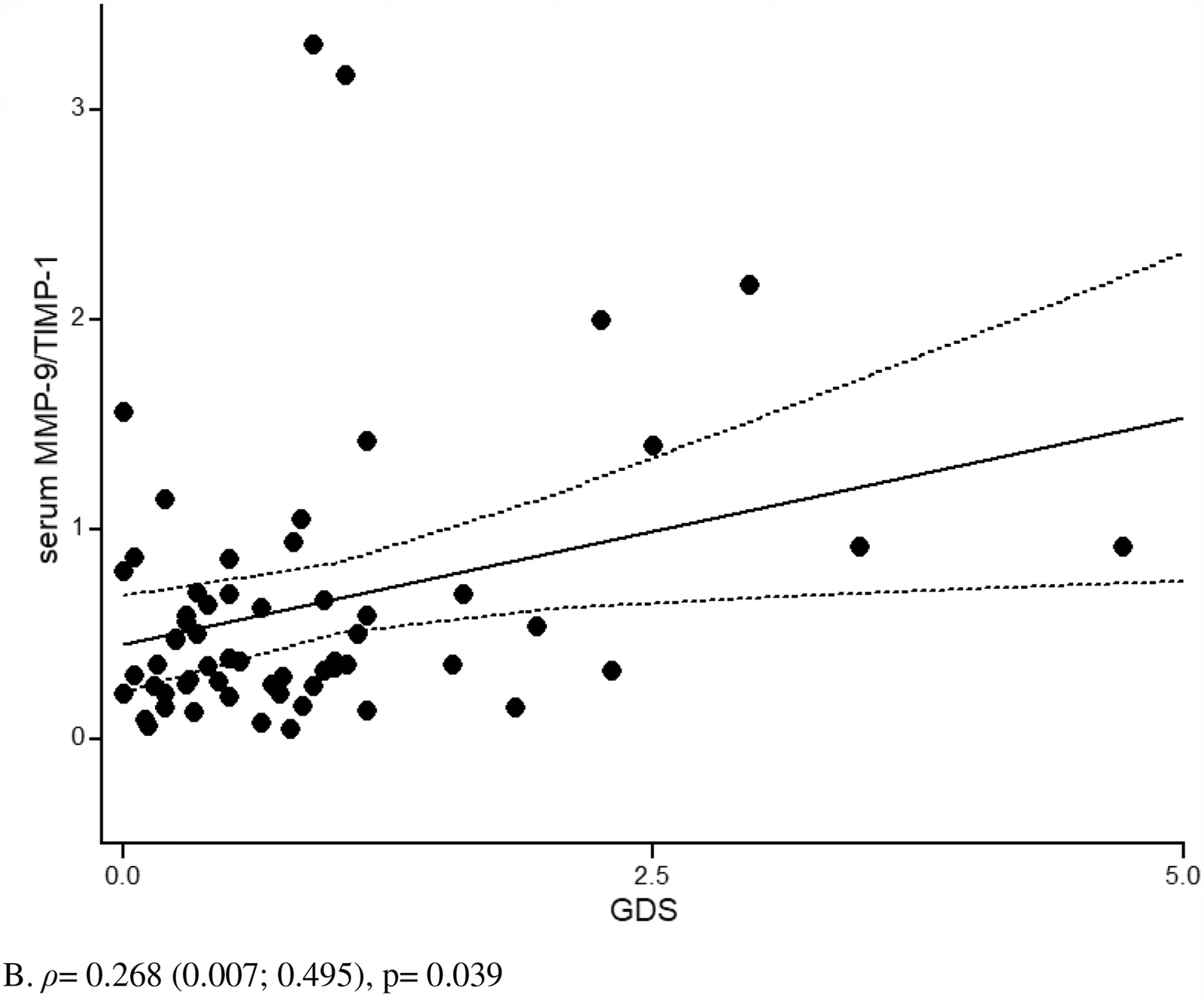

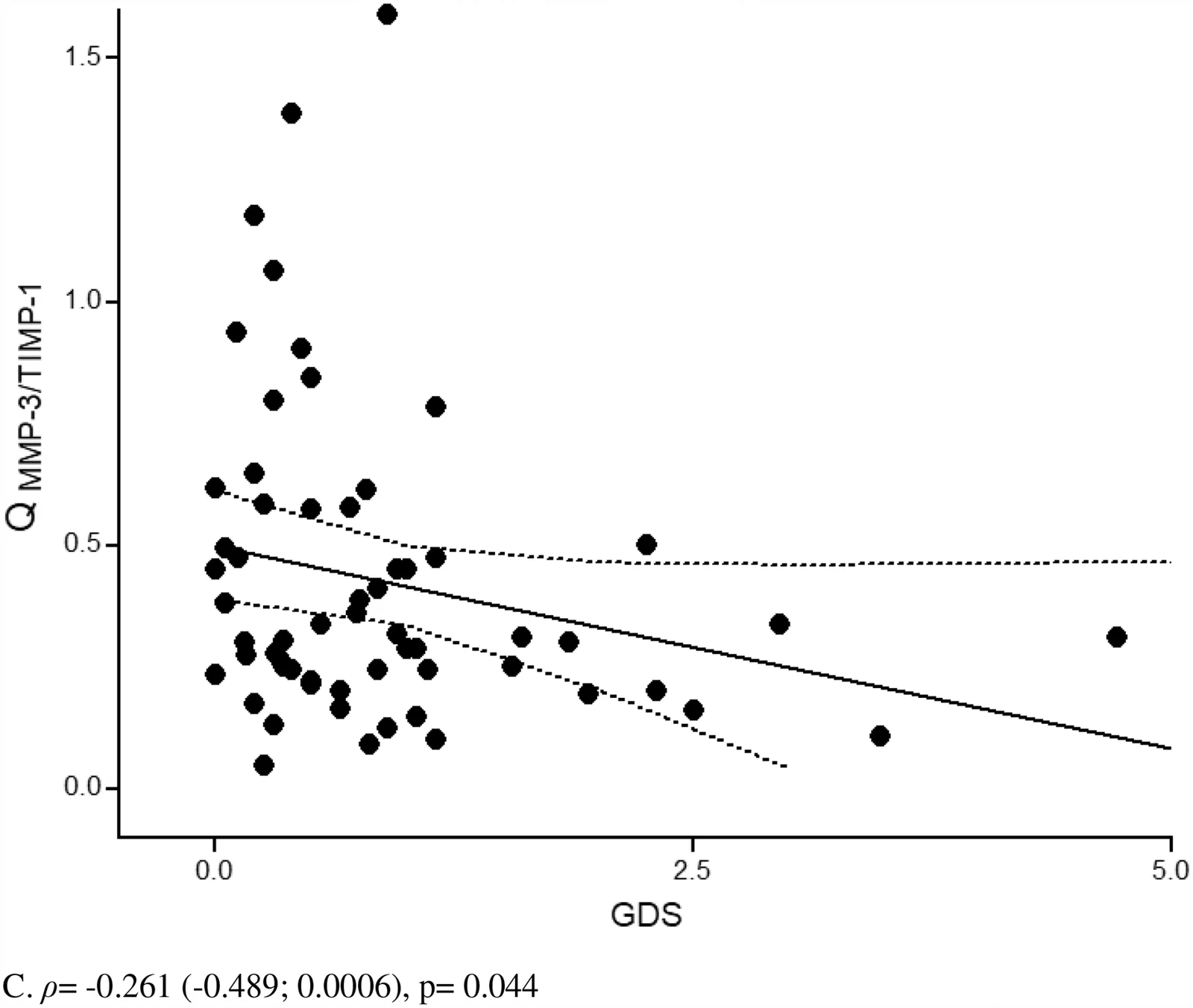

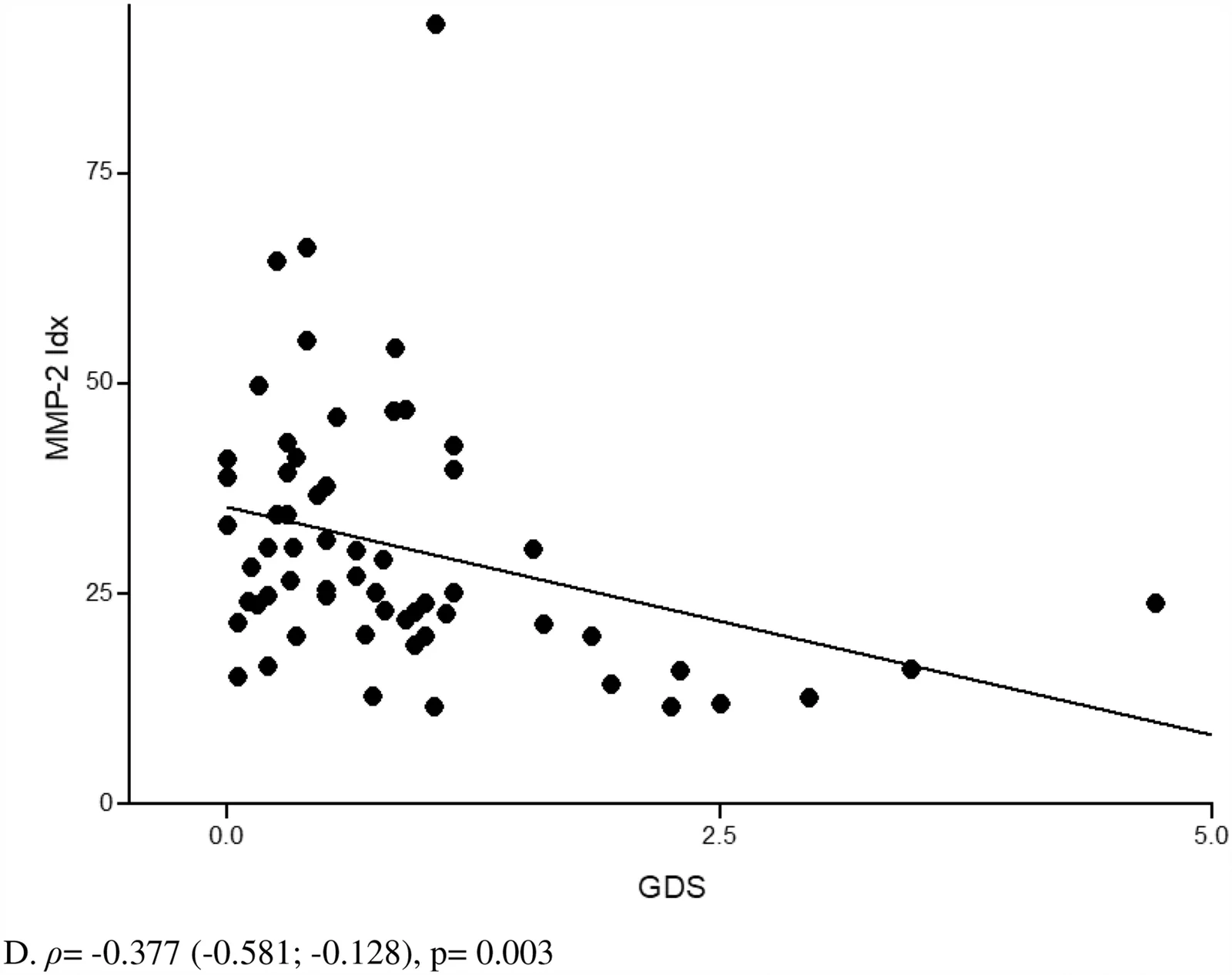

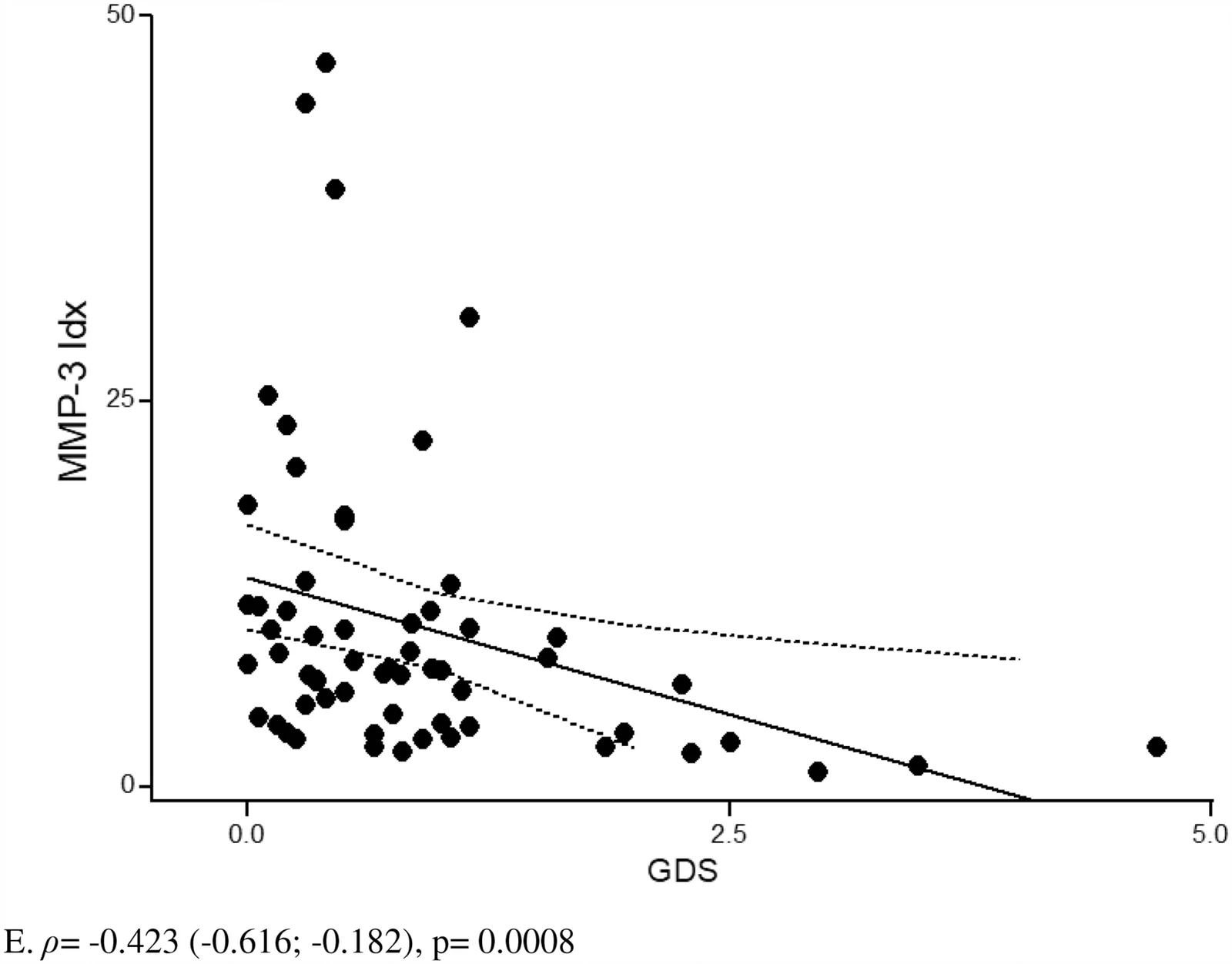

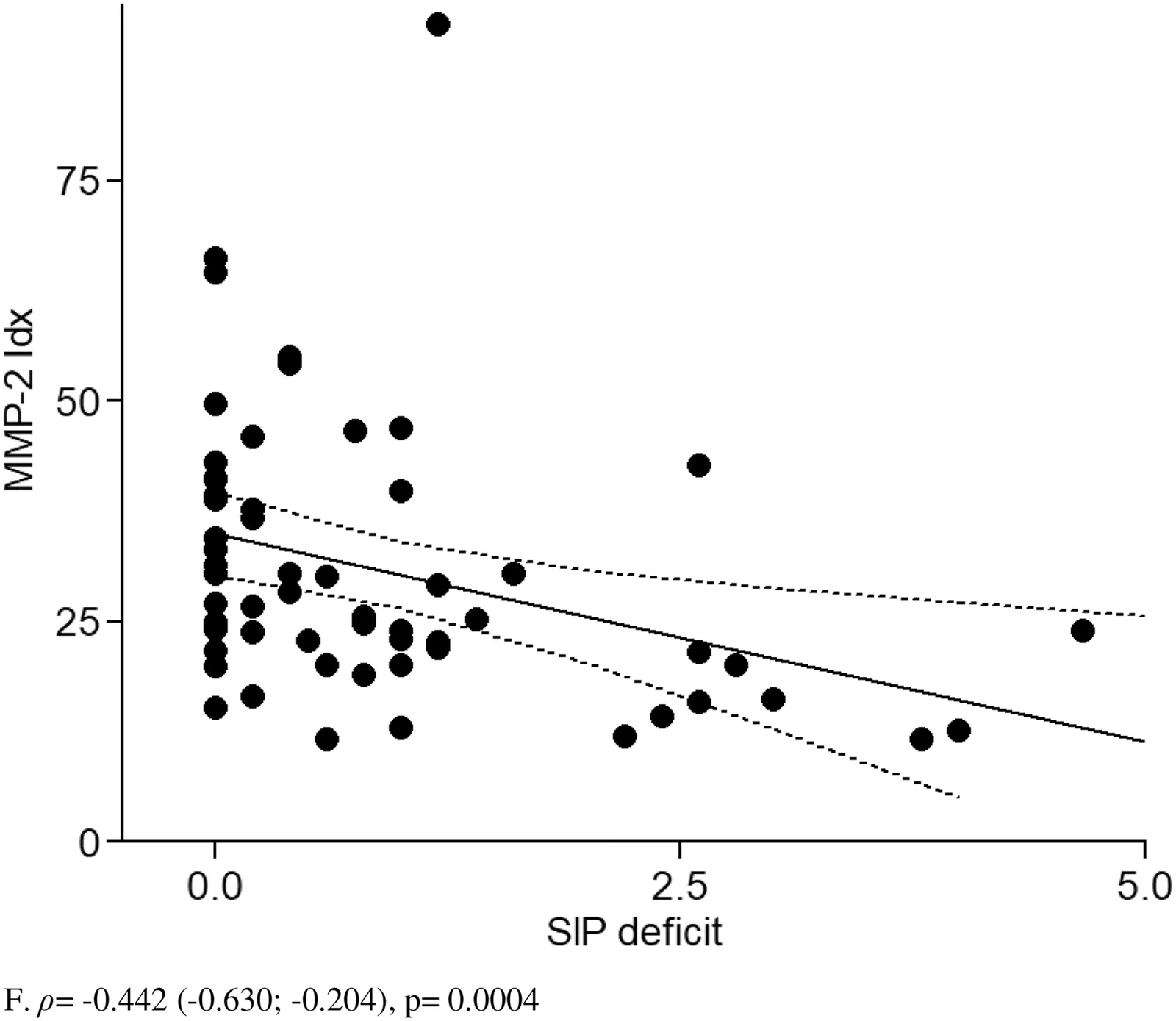

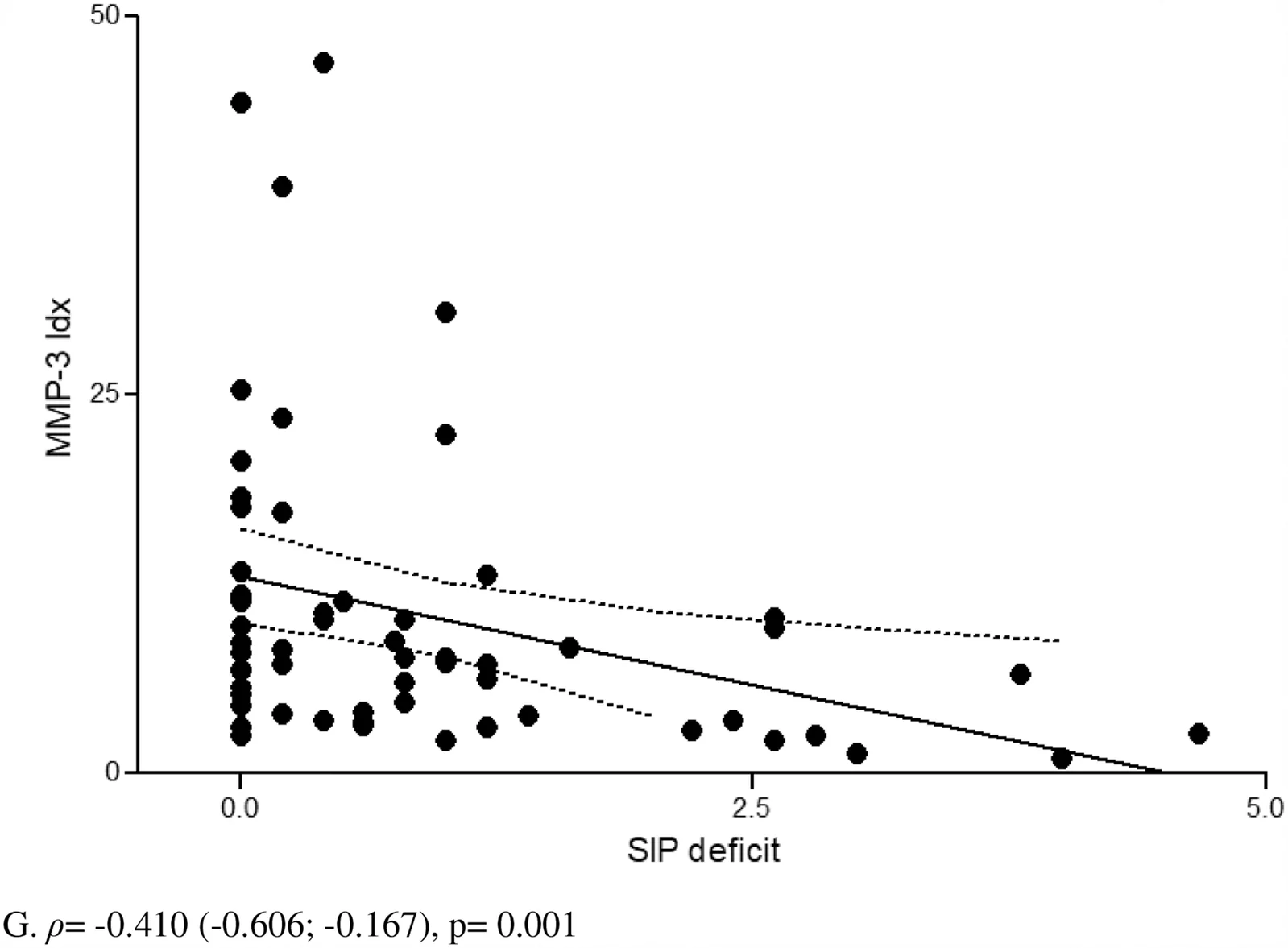

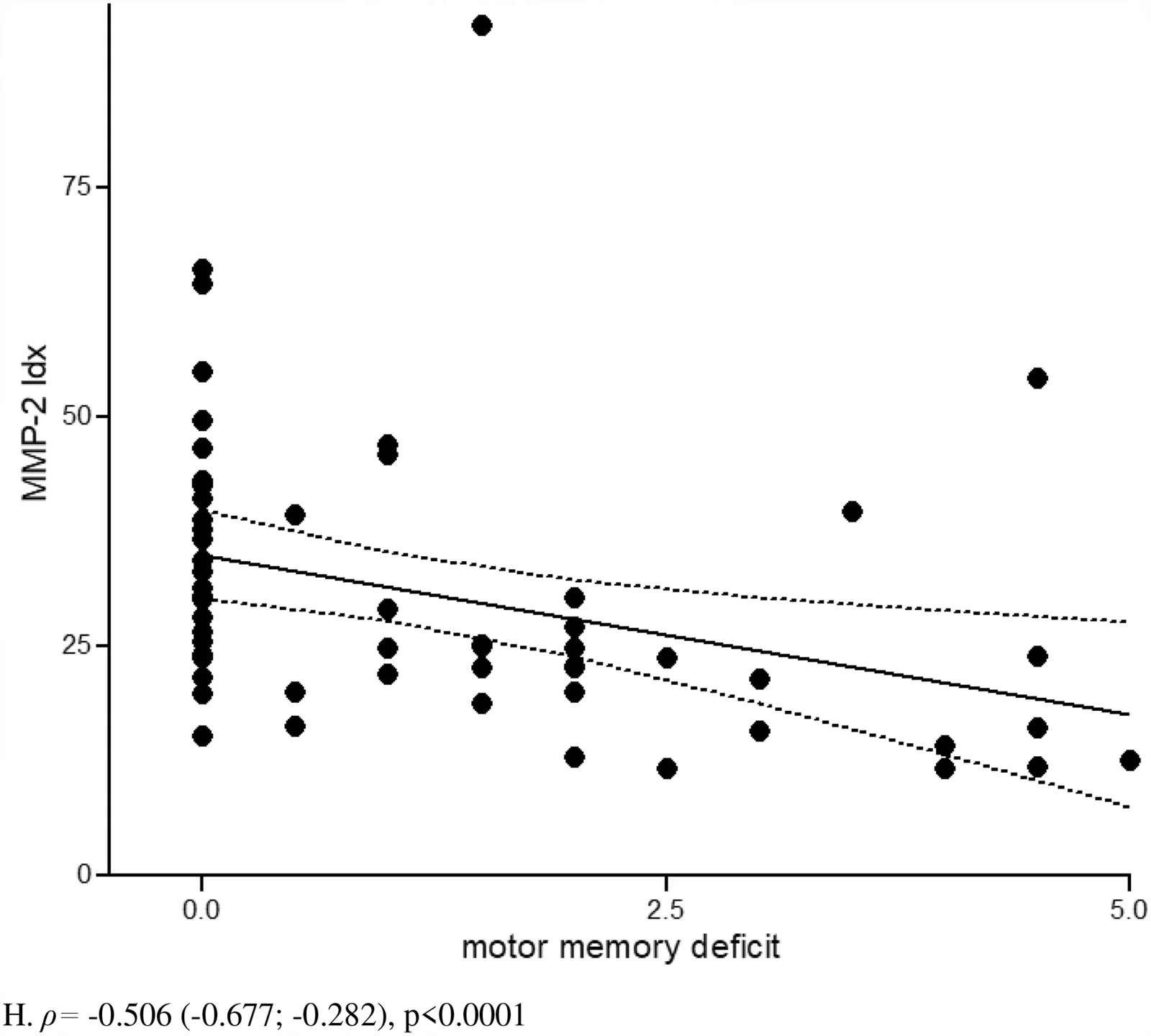

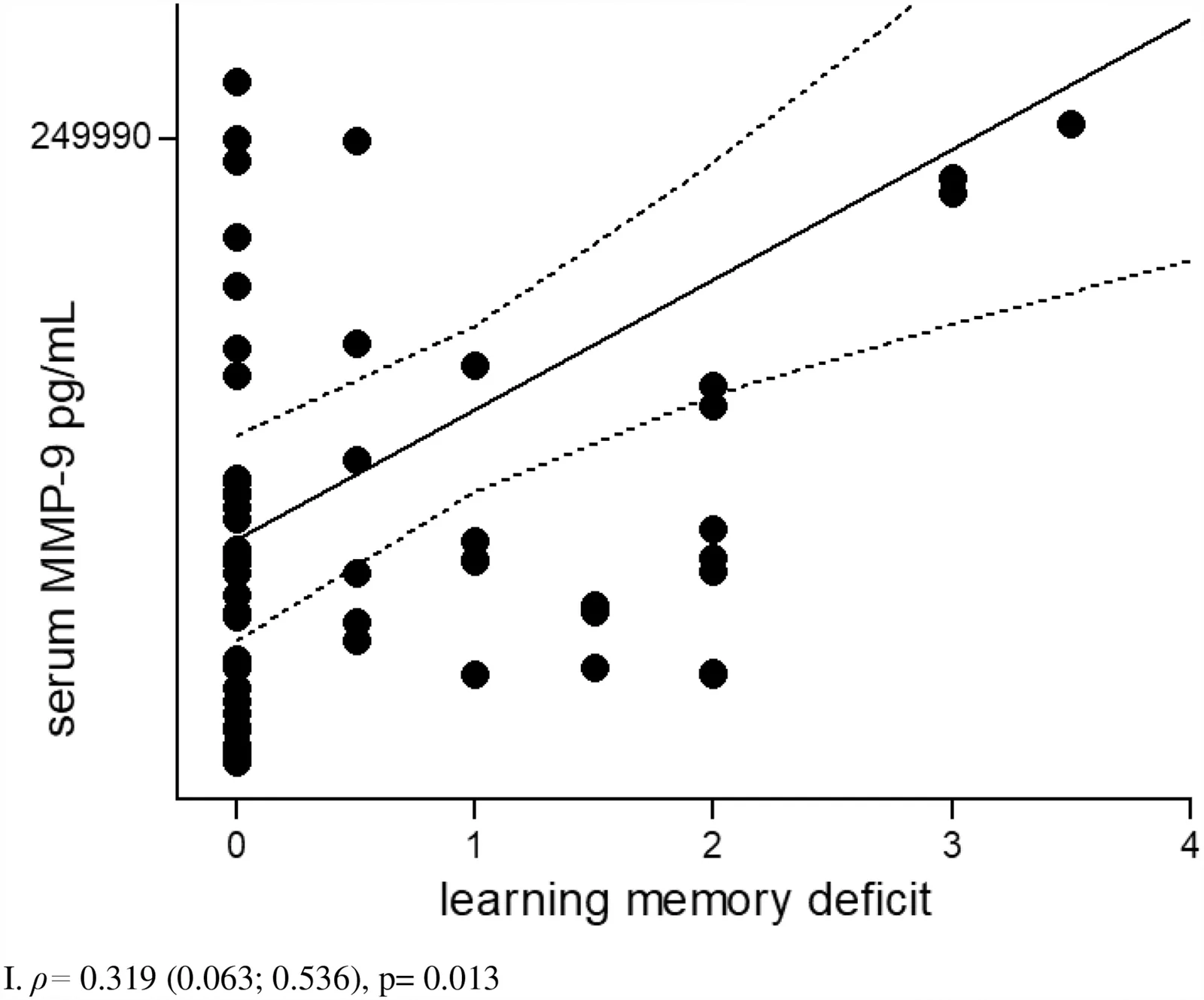
Scatterplots of the correlation between CSF MMP and TIMP with cognitive domains and global deficit score Correlation coefficients were estimated using Spearman’s rank-order method. Values are presented as Spearman’s rank correlation coefficients (*ρ*), 95% confidence intervals, and p values. MMP, metalloproteinases; TIMP, tissue inhibitor of metalloproteinases; CSF, cerebrospinal fluid; GDS, global deficit score. A. CSF MMP-9 pg/ mL versus working memory; B. serum MMP-9/TIMP-1 versus GDS; C. Q_MMP-3/TIMP-1_ versus GDS; D. MMP-2 Index versus GDS; E. MMP-3 Index versus GDS; F. MMP-2 Index versus SIP deficit; G. MMP-3 Index versus SIP deficit; H. MMP-2 Index versus motor memory deficit; I. serum MMP-9 pg/mL versus learning memory deficit. MMP Index = (CSF MMP × albumin serum) / (serum MMP × albumin CSF).

**Figure 3.**
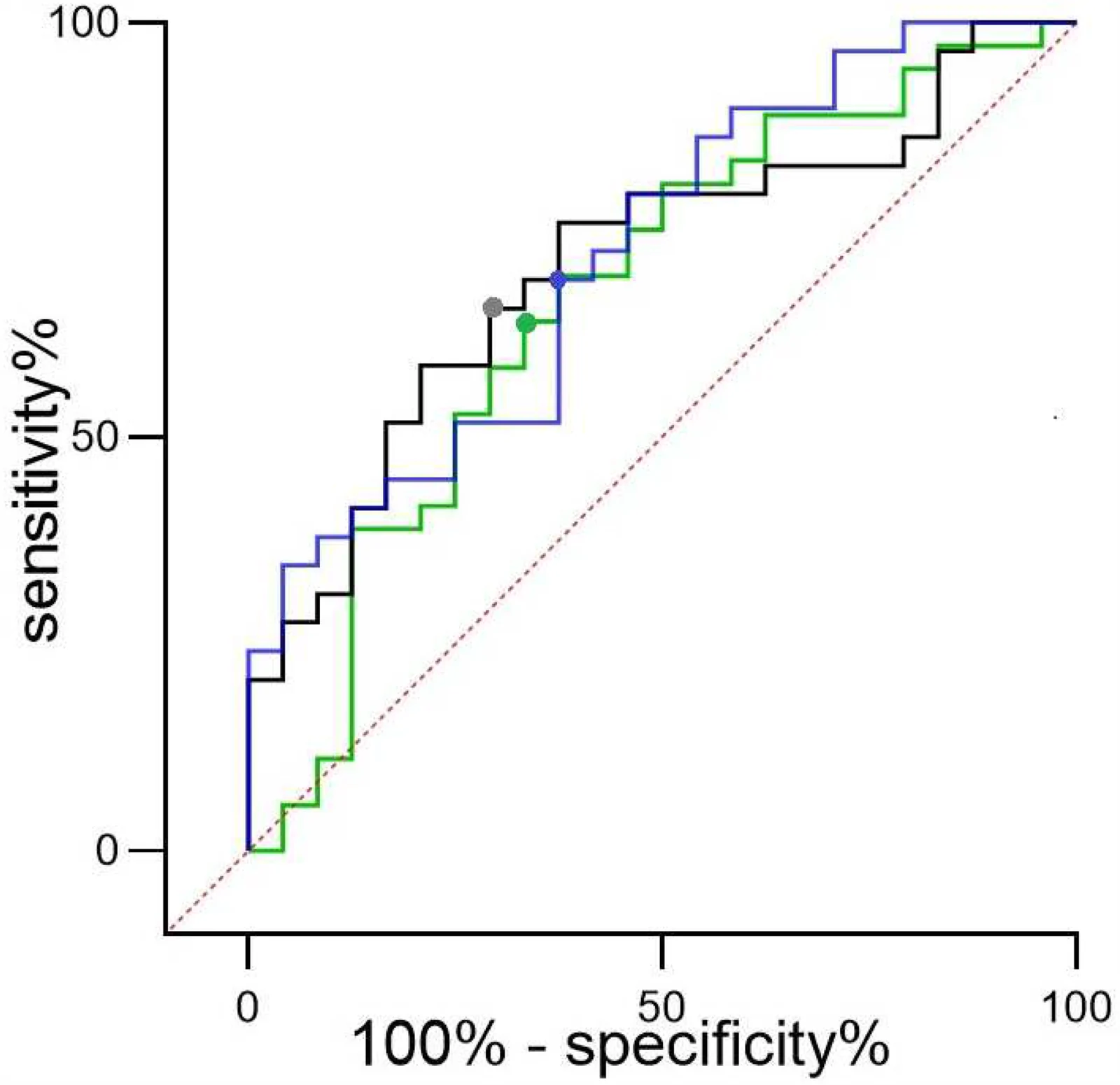
Receiver operating characteristic (ROC) curve to evaluate predictive characteristics of serum MMP3/TIMP-1, MMP-2 Index, and MMP-3 Index for the HAND diagnosis Serum MMP3/TIMP-1 (green line); area under the curve (AUC) = 0.671 (95% CI, 0.527–0.816), p= 0.026; optimal cut-off point >0.04. For MMP-2 Index (black line); AUC= 0.711 (0.572;0.852), p= 0.009; optimal cut-off point <26.91. For MMP-3 Index (blue line): AUC= 0.721 (0.585; 0.857), p= 0.006; optimal cut-off point <7.63. For serum MMP3/TIMP, the receiver operating characteristic (ROC) curve was generated by comparing the PWH subgroup with a Global Deficit Score (GDS) ≥ 0.5 to the PWH subgroup with a GDS below 0.5, which served as the reference standard. For the MMP2 and MMP-3 Index, the comparison was made between the HAND Frascati classification groups, specifically asymptomatic individuals versus those with normal neurocognitive evaluation, as the gold standard. The dot represents the optimal cut-off point, and the dashed red line is the chance line. The AUC acts as a single measure, independent of prevalence, which summarizes the discriminative ability of a test across the full range of cutoffs, where the higher the AUC is, the better the test will be. A perfect test would have an AUC of 1.0, while a completely ineffective test (where the curve falls on a diagonal line) has an AUC of 0.5. The AUC of 0.7 to 0.8 indicates a diagnostic test is acceptable with good discrimination. MMP Index = (CSF MMP × albumin serum) / (serum MMP × albumin CSF).

**Table 1.** Levels of MMP and TIMP in CSF, serum, CSF/serum quotient, and MMP indexes in individuals with GDS ≥0.50 versus GDS <0.50

	CSF				SERUM				CSF/serum			
	GDS ≥0.50 n= 36	GDS <0.50 n= 24	Coheńs d (95%CI)	p	GDS ≥0.50 n= 36	GDS <0.50 n= 24	Coheńs d (95%CI)	p	GDS ≥0.50 n= 36	GDS <0.50 n= 24	Coheńs d (95%CI)	p
MMP-2 pg/mL	13700(11180; 16820)	13800(10450; 18830)	0.093 (−0.444, 0.63)	0.900	70500(65970; 86240)	68160(58990; 78340)	0.358 (0.179, 0.895)	0.26	0.188 (0.147; 0.235)	0.208 (0.163; 0.294)	−0.104 (0.641, 0.433)	0.71
MMP-3 pg/mL	517.5 (355.0; 651.0)	543.5 (380.0; 684.0)	−0.152 (−0.689, 0.384)	0.900	11480 (7722; 15390)	7889 (4187; 12820)	0.607 (0.07, 1.144)	**0.031/0.098 [1]**	0.047 (0.033; 0.072)	0.064 (0.035; 0.136)	−0.665 (−1.202, −0.129)	**0.018/0.120 [1]**
MMP-9 pg/mL	135.5 (71.76; 389.5)	120.5 (53.13; 745.5)	−0.033 (−0.57, 0.504)	0.900	109600 (67400; 232700)	93210 (64910; 151700)	0.524 (0.013, 1.061)	**0.061/0.120 [1]**	0.0015 (0.0003; 0.0046)	0.0016 (0.0004; 0.0077)	−0.218 (−0.755, 0.319)	0.613
TIMP-1 pg/mL	37040 (29120; 46400)	40540 (30700; 55960)	−0.368 (−0.905, 0.169)	0.490	249800 (185000; 282900)	258200 (224000; 291900)	−0.363 (0.899, 0.174)	0.26	0.153 (0.113; 0.214)	0.167 (0.127; 0.222)	−0.137 (−0.674, 0.4)	0.670
TIMP-2 pg/mL	48750 (37000; 58350)	54000 (44470; 66120)	−0.368 (−0.905, 0.169)	0.490	122400 (107600; 139000)	125700 (106700; 139700)	0.016 (−0.52, 0.553)	0.950	0.407 (0.337; 0.468)	0.432 (0.372; 0.609)	−0.393 (−0.93, 0.144)	0.290
MMP-9/TIMP-1	0.004 (0.002; 0.011)	0.004 (0.002; 0.018)	0.074 (−0.462, 0.611)	0.900	0.445 (0.283; 0.919)	0.351 (0.237; 0.617)	0.586 (0.049, 1.123)	**0.037/0.098 [1]**	0.011(0.003; 0.034)	0.010 (0.003; 0.046)	−0.203 (−0.74, 0.334)	0.60
MMP-2/TIMP-2	0.303 (0.258; 0.344)	0.279 (0.189; 0.353)	0.074 (−0.462, 0.611)	0.490	0.614 (0.501; 0.742)	0.554 (0.468; 0.665)	0.281 (0.256, 0.817)	0.354	0.499 (0.372; 0.612)	0.484 (0.354; 0.587)	0.178 (−0.358, 0.715)	0.61
MMP-3/TIMP-1	0.013 (0.009; 0.020)	0.012 (0.010; 0.020)	0.195 (−0.342, 0.732)	0.900	0.048 (0.029; 0.068)	0.028 (0.018; 0.049)	0.811 (0.274, 1.348)	**0004/0.036 [1]**	0.308 (0.203; 0.453)	0.416 (0.260; 0.724)	−0.562 (−1.099, −0.026)	**0.045/0.14 [1]**
MMP-2 Idx									23.97 (19.48; 30.93)	33.84 (24.54; 41.20)	−0.538 (−1.075, −0.001)	**0.055/0.14 [1]**
MMP-3 Idx									6.947 (3.135; 9.955)	10.000 (6.291; 19.500)	−0.8 (−1.337, −0.263)	**0.005/0.065 [1]**
MMP-9 Idx									0.205 (0.065; 0.724)	0.335 (0.094; 1.280)	−0.354 (−0.891, 0.183)	0.33

Values represent median (interquartile range). GDS, global deficit score; MMP, metalloproteinases; TIMP, tissue inhibitor of metalloproteinases; CSF, cerebrospinal fluid.

Plasma HIV viral load suppression and nadir CD4 count were included in models as covariates. *p*-values were adjusted for multiple testing of multiple related biomarkers using the Benjamini-Hochberg (BH) procedure, [1] *p* before and after adjustment for multiple testing of related biomarkers. Group differences are presented as Cohen’s d; CI = confidence interval.

Significant differences are in **bold** typeface.

MMP index (Idx)= (CSF MMP × serum albumin)/ (serum MMP ×CSF albumin)

**Table 2.** Levels of CSF MMP and TIMP in PWoH and the three HAND subgroups

CSF	PWoH n= 19	A-NP-NML n= 24	B-ANI n= 29	C-HAD n= 5 [1]	A×B×C p	A × PWoH	B × PWoH	C × PWoH
MMP-2 pg/mL	9279 (8378; 10450)	13800 (10450; 18830)	13000 (11060; 17700)	14860 (11720; 16320)	0.85	**0.0007**	**<0.0001**	**0.0038**
MMP-3 pg/mL	431.00 (349.00; 550.50)	543.50 (380.0; 684.0)	529.0 (355.0; 651.0)	493.0 (278.0; 721.0)	0.85	0.1491	0.1912	0.4995
MMP-9 pg/mL	14.64 (7.76; 17.13)	120.50 (53.13; 745.50)	140.0 (72.25; 577.0)	79.15 (56.17; 1249)	0.85	**<0.0001**	**<0.0001**	**0.0029**
TIMP-1 pg/mL	34770 (29060;48470)	40540 (30700; 55960)	37250 (28700; 46400)	32010 (28680; 54880)	0.85	0.3465	0.8166	1.0000
TIMP-2 pg/mL	44020 (36140;66070)	54000 (44470; 66120)	50050 (37000; 61440)	41960 (34120; 53860)	0.85	0.1908	0.8003	0.8870
MMP-9/TIMP-1	0.0003 (0.0002;0.0006)	0.004 (0.002; 0.018)	0.004 (0.002; 0.013)	0.003 (0.001; 0.028)	0.85	**<0.0001**	**<0.0001**	**0.0038**
MMP-2/TIMP-2	0.155 (0.130; 0.281)	0.278 (0.189; 0.353)	0.294 (0.234; 0.356)	0.335 (0.293; 0.355)	0.85	**0.0067**	**0.0008**	**0.0098**
MMP-3/TIMP-1	0.012 (0.008; 0.015)	0.012 (0.010; 0.020)	0.013 (0.008; 0.021)	0.009 (0.009; 0.024)	0.85	0.5491	0.1985	0.7223

Values represent median (interquartile range) or [1] median (minimum; maximum). The three HAND subgroups were compared using ANCOVA, adjusting for HIV RNA and nadir CD4, and pairwise comparison with Tukey’s HSD test. Multiple testing correction was performed with the Benjamini Hochberg (BH) procedure. MMP, metalloproteinases; TIMP, tissue inhibitor of metalloproteinases; CSF, cerebrospinal fluid. The Cohen’s d (95%CI) of the significant comparison is shown in Suppl Table 2. The three HAND subgroups include (A) neuropsychologically normal- NP-NML, (B) asymptomatic neurocognitive impairment- ANI, (C) HIV-associated dementia- HAD.

The MND group was not included in the analyses due to the small number of cases (n = 2).

**Table 3.** Levels of serum MMP and TIMP in PWoH and the three HAND subgroups

SERUM	PWoH n= 19	A-NP-NML n= 24	B-ANI n= 29	C-HAD n= 5 [1]	A×B×C p value	A × PWoH	B × PWoH	C × PWoH
MMP-2 pg/mL	71590 (60860; 77470)	68160 (58990; 78340)	69790 (65950; 86320)	68130 (59980; 75900)	0.65	0.9894	0.5240	0.8755
MMP-3 pg/mL	9268 (3065; 16470)	7889 (4187; 12820)	11490 (6404; 15120)	9519 (5485; 17080)	0.24	0.7047	0.1518	0.5224
MMP-9 pg/mL	79190 (57030; 121300)	93210 (64910; 151700)	105600 (59020; 232700)	88910 (51750; 724300)	0.19	0.4668	0.1869	0.3888
TIMP-1 pg/mL	209500 (183700; 255600)	258200 (224000; 291900)	253400 (185000; 282900)	218600 (176200; 332500)	0.65	**0.0086**	**0.0551**	0.5224
TIMP-2 pg/mL	122400 (105700; 141800)	125700 (106700; 139700)	126200 (107600; 141200)	119100 (100000; 162400)	0.92	0.7785	0.7518	0.8870
MMP-9/TIMP-1	0.457 (0.261; 0.597)	0.351 (0.237; 0.617)	0.501 (0.258; 0.919)	0.360 (0.156; 3.313)	0.056/0.19[2]	0.6817	0.5240	0.6383
MMP-2/TIMP-2	0.566 (0.512; 0.650)	0.554 (0.468; 0.665)	0.613 (0.493; 0.763)	0.585 (0.369; 0.681)	0.67	0.4998	0.8377	1.0000
MMP-3/TIMP-1	0.038 (0.013; 0.075)	0.028 (0.018; 0.049)	0.047 (0.026; 0.069)	0.037 (0.029; 0.070)	0.027/0.19 [2]	0.6510	0.5134	0.7223

Values represent median (interquartile range) or [1] median (minimum; maximum). The three HAND subgroups were compared using ANCOVA, adjusting for HIV RNA and nadir CD4, and pairwise comparison with Tukey’s HSD test. Multiple testing correction was performed with the Benjamini Hochberg (BH) procedure. [2] p-value/the BH-adjusted p-value; MMP, metalloproteinases; TIMP, tissue inhibitor of metalloproteinases; CSF, cerebrospinal fluid. The Cohen’s d (95%CI) for the significant comparisons: TIMP-1 in A versus PWoH 0.910 (0.278; 1.542); B versus PWoH 0.617 (0.025; 1.208).

The three HAND subgroups include: (A) neuropsychologically normal- NP-NML, (B) asymptomatic neurocognitive impairment- ANI, (C) HIV-associated dementia- HAD. The MND group was not included in the analyses due to the small number of cases (n = 2).

**Table 4.** Levels of MMP and TIMP in CSF/serum quotient (Q) and index (Idx) in PWoH and the three HAND subgroups

	PWoH n= 19	A-NP-NML n= 24	B-ANI n= 29	C-HAD n= 5 [1]	A×B×D p	A × PWoH	B × PWoH	C × PWoH
**Q**_**MMP-2**_	0.139 (0.109; 0.175)	0.208 (0.163; 0.294)	0.179 (0.142; 0.245)	0.225 (0.172; 0.235)	0.98	**0.001**	**0.004**	**0.008**
**Q**_**MMP-3**_	0.065 (0.034; 0.158)	0.064 (0.035; 0.136)	0.048 (0.032; 0.075)	0.033 (0.029; 0.131)	0.21	0.990	0.238	0.286
**Q**_**MMP-9**_	0.00009 (0.0002; 0.0004)	0.0016 (0.0004; 0.0077)	0.0015 (0.0003; 0.0082)	0.0015 (0.00008; 0.0162)	0.49	**0.0001**	**<0.0001**	0.137
**Q**_**TIMP-1**_	0.199 (0.135; 0.243)	0.167 (0.127; 0.222)	0.152 (0.110; 0.213)	0.168 (0.096; 0.251)	0.93	0.517	0.135	0.477
**Q**_**TIMP-2**_	0.424 (0.330; 0.574)	0.432 (0.372; 0.609)	0.419 (0.331; 0.505)	0.351 (0.258; 0.435)	0.48	0.549	0.784	0.394
**Q**_**MMP-9/TIMP-1**_	0.0009(0.0005;0.002)	0.010 (0.003; 0.046)	0.012 (0.003; 0.057)	0.015 (0.0003; 0.076)	0.48	**<0.0001**	**<0.0001**	0.100
**Q**_**MMP-2/TIMP-2**_	0.276 (0.223; 0.472)	0.484 (0.354; 0.587)	0.448 (0.364; 0.638)	0.525(0.504; 0.908)	0.75	**0.0014**	**0.0017**	0.015
**Q**_**MMP-3/TIMP-1**_	0.333 (0.206; 0.718)	0.416 (0.260; 0.724)	0.319 (0.199; 0.464)	0.290 (0.128; 0.784)	0.39	0.566	0.736	0.570
**MMP-2 Idx**	30.16 (23.90; 40.04)	**33.840 (24.540; 41.200) [3]**	**22.980 (16.050; 30.810) [3]**	30.440 (20.050; 92.680)	**0.047/0. 15 [2]**	0.625	**0.0406**	1.000
**MMP-3 Idx**	16.860 (7.592; 36.84)	**10.000 (6.291; 19.500) [3]**	**6.606 (3.037; 9.237) [3]**	8.389 (2.605; 30.450)	**0.012/0.15[2]**	0.236	**0.0013**	0.286
**MMP-9 Idx**	0.0348 (0.018; 0.100)	0.335 (0.094; 1.280)	0.230 (0.061; 0.786)	0.136 (0.007; 2.520)	0.48	**0.0003**	**0.0015**	0.159

Values represent median (interquartile range) or [1] median (minimum; maximum). The three HAND subgroups were compared using ANCOVA, adjusting for HIV RNA and nadir CD4, and pairwise comparison with Tukey’s HSD test. Multiple testing correction was performed with the Benjamini Hochberg (BH) procedure. [2] p-value/the BH-adjusted p-value; significant differences are in **bold**; Cohen’s d (95% CI) of the significant pairwise comparisons were shown in Suppl. Table 3.

MMP, metalloproteinases; TIMP, tissue inhibitor of metalloproteinases; CSF, cerebrospinal fluid.

The three HAND subgroups include: (A) neuropsychologically normal- NP-NML, (B) asymptomatic neurocognitive impairment- ANI, (C) HIV-associated dementia- HAD.

MMP Index = (CSF MMP × albumin serum)/ (serum MMP × albumin CSF).

[3] pairwise comparison with Tukey test: A versus B: MMP-2 Idx, p= 0.022, Cohen’s d (95% CI) −0.747 (−1.307; −0.188); MMP-3 Idx, p= 0.008, −0.805 (−1.367; −0.243). The MND group was not included in the analyses due to the small number of cases (n = 2).

## Data Availability

Anonymized data from the current study will be made available at the request of qualified investigators, if approved by our Research Ethics Board.
